# Risk Factors Underlying COVID-19 Lockdown-Induced Mental Distress

**DOI:** 10.3389/fpsyt.2020.603014

**Published:** 2020-12-21

**Authors:** Jan Sebastian Novotný, Juan Pablo Gonzalez-Rivas, Šárka Kunzová, Mária Skladaná, Anna Pospíšilová, Anna Polcrová, Jose Ramon Medina-Inojosa, Francisco Lopez-Jimenez, Yonas Endale Geda, Gorazd Bernard Stokin

**Affiliations:** ^1^Translational Neuroscience and Aging Program, Centre for Translational Medicine, International Clinical Research Centre, St. Anne's University Hospital, Brno, Czechia; ^2^Kardiovize Study, International Clinical Research Centre, St. Anne's University Hospital, Brno, Czechia; ^3^Department of Global Health and Population, Harvard TH Chan School of Public Health, Harvard University, Boston, MA, United States; ^4^Division of Preventive Cardiology, Department of Cardiovascular Medicine, Mayo Clinic, Rochester, MN, United States; ^5^Department of Neurology, Barrow Neurological Institute, Phoenix, AZ, United States; ^6^Translational Neuroscience and Aging Program, Mayo Clinic, Rochester, MN, United States; ^7^Division of Neurology, University Medical Centre, Ljubljana, Slovenia

**Keywords:** COVID-19, stress levels, depressive symptoms, risk factors, feeling of loneliness

## Abstract

Recent reports suggest that the COVID-19 lockdown resulted in changes in mental health, however, potential age-related changes and risk factors remain unknown. We measured COVID-19 lockdown-induced stress levels and the severity of depressive symptoms prior to and during the COVID-19 lockdown in different age groups and then searched for potential risk factors in a well-characterized general population-based sample. A total of 715 participants were tested for mental distress and related risk factors at two time-points, baseline testing prior to COVID-19 and follow-up testing during COVID-19, using a battery of validated psychological tests including the Perceived Stress Scale and the Patient Health Questionnaire. Longitudinal measurements revealed that the prevalence of moderate to high stress and the severity of depressive symptoms increased 1.4- and 5.5-fold, respectively, during the COVID-19 lockdown. This surge in mental distress was more severe in women, but was present in all age groups with the older age group exhibiting, cross-sectionally, the lowest levels of mental distress prior to and during the lockdown. Illness perception, personality characteristics such as a feeling of loneliness, and several lifestyle components were found to be associated with a significant increase in mental distress. The observed changes in mental health and the identified potential risk factors underlying these changes provide critical data justifying timely and public emergency-tailored preventive, diagnostic, and therapeutic mental health interventions, which should be integrated into future public health policies globally.

## Introduction

The novel coronavirus disease 2019 (COVID-19) outbreak in Wuhan, China, evolved rapidly into a pandemic with businesses, governments, and international organizations taking unprecedented action to limit the threat to global health. At an individual level, the COVID-19 pandemic presented several challenges ranging from fear of infection by a poorly understood illness with unclear prognosis to the limited possibilities of diagnostics and a shortage of personal protection equipment (1). At a societal level, actions to curb the spread of COVID-19 led to the implementation of unfamiliar public health measures such as social isolation and distancing, remote education and work, and a ban on travel (2). Several of these measures have already been reported to influence mental health in the general population in previous outbreaks (3–5). For example, up to 33% of surveyed participants reported increased worries during the swine flu outbreak in the UK (6), 48% of the general population exhibited depressive symptoms during the Ebola outbreak in Sierra Leone (7), and 57% of subjects reported increased irritability during the severe acute respiratory syndrome (SARS) outbreak in Hong Kong (8). Although these outbreaks were geographically limited compared with COVID-19, findings from these outbreaks are consistent with the earliest studies evaluating the impact of the COVID-19 lockdown on mental health. These studies largely estimated the frequency of the diverse components of mental distress cross-sectionally either in specific populations such as healthcare professionals (9–11) or in convenience samples from regions of the world that were at the forefront of the COVID-19 pandemic, such as China (12, 13) and Italy (14). More recently, longitudinal studies examining the impact of COVID-19 on mental health have started to emerge (Table 1, Supplementary Figure 1). These studies most commonly assessed general mental distress at the time (26–30) or addressed specific symptoms of mental distress by comparing COVID-19 with pre-COVID-19 data obtained largely from national survey-based probability samples (17, 19–21, 31). Collectively, these studies showed significantly increased mental distress in response to COVID-19. Relatively few studies to date, however, addressed age-related changes and investigated risk factors associated with COVID-19-induced mental distress (32, 33). As a result, the mechanisms underlying the development of mental distress in response to COVID-19 remain poorly understood. To address this gap, we took advantage of a well-characterized general population-based sample representing randomly selected 1% of the population of the city of Brno, Czech Republic, randomly selected, to critically measure changes in mental health during the COVID-19-induced lockdown in order to probe for age-related changes and potential risk factors.

**Table 1:** Review of current longitudinal studies on the impact of COVID-19 on mental health.

**Authors**	**Country**	**Sample size (N)**	**Source of pre-COVID-19 data**	**Source of COVID-19 data**	**Main impact of COVID-19 on mental health**
Shanahan et al. (15), Psychol Med	Switzerland	768	Zurich project on the social development	Online survey	Increase in mental distress
Brailovskaia et al. (16), Int J Clin Heal Psychol	Germany	436	Bochum optimism and mental health project	Online survey	Stress affects COVID-19 response
McGinty et al. (17), JAMA	USA	35,000	NORC's AmeriSpeak panel	Online survey	Increase of mental distress
Van der Velden et al. (18), J Affect Disord	Netherland	3,983	Dutch longitudinal population-based LISS panel	Online survey	No change in mental distress
Pierce et al. (19), The Lancet Psychiatry	UK	17,452	UK household longitudinal study	Online survey	Increase of mental distress
Niedzwiedz et al. (20), medRxiv	UK	9,748	UK household longitudinal survey	Online survey	Increase of mental distress
Chandola et al. (21), medRxiv	UK	17,452	UK household longitudinal survey	Online survey	Higher incidence of common mental disorder
Daly et al. (22), Psychol Med	UK	12,074	UK household longitudinal survey	Online survey	Increase of mental health problems
Kwong et al. (23), medRxiv	UK	10659	Avon longitudinal study of parents and children and generation Scotland: Scottish family health study	Online survey	Increased anxiety
Kim et al. (24), medRxiv	South Africa	221	Developmental pathways for health research unit epidemiological surveillance study	Phone survey	Predicted greater depressive symptoms
Biddle et al. (25)	Australia	1,745	ANUpoll	Online survey	Increase of mental distress

## Materials and Methods

### Study Design and Sample

A summary of the Kardiovize study baseline examination protocol and characteristics of the general population-based sample have been published previously (34). In brief, the Kardiovize study is a prospective longitudinal epidemiological cohort that investigates health-related topics in Central Europe carried out on a representative randomly selected 1% population sample of the residents of the city of Brno, Czech Republic. Between March 16 and May 17, 2020, the Czech Republic implemented a strict public lockdown in response to the COVID-19 pandemic, which included national quarantine with the closure of schools, shops (except for daily essentials), restaurants, and borders, social distancing, and the obligatory use of personal protection equipment. At the beginning of the COVID-19 outbreak in Europe, the COVID-19 add-on study protocol, including a custom designed e-questionnaire, was promptly prepared. Its purpose was to measure changes in mental health during the COVID-19 lockdown and to identify potential risk factors underlying these changes. The original COVID-19 add-on study was conducted from April 24 to May 27, 2020.

### Selection Procedure

The inclusion criteria for the COVID-19 add-on study were all participants of the Kardiovize study with available baseline data on stress and depressive symptoms (Supplementary Figure 2). Those diagnosed with a COVID-19 infection (two cases) were excluded. A total of 1,823 Kardiovize study participants were invited electronically to join the COVID-19 add-on study. An e-questionnaire was completed by 715 participants in roughly 4 weeks through an online survey module using a validated RedCap software tool (35). The e-questionnaire consisted of several items (see section Measures and Instruments), including the Perceived Stress Scale (36) (PSS) and the Patient Health Questionnaire (37) (PHQ), which were also assessed during the baseline measurements of the original Kardiovize study in previous years.

### Measures and Instruments

The e-questionnaire measured general demographics (sex, age, education, and marital status), including questions on how the COVID-19 lockdown affected participant's lifestyle, their experience with the COVID-19 lockdown, as well as their current medical status (Supplementary Material). Several psychological questionnaires evaluating stress, depressive symptoms, illness perception, and loneliness were also included. In brief, the presence and severity of stress was assessed using PSS with a scale ranging from 0 to 40. Stress levels were categorized as low (score of 0–13), medium (score of 14–26), or high (score of 27–40). The presence and severity of depressive symptoms was assessed using the identical two items from the PHQ-9 (prior to COVID-19) and the PHQ-4 (38) (during COVID-19) with a scale range of 0–6. Depressive symptoms were considered present if the sum of the score of the two PHQ items were ≥3 (38). The perception of COVID-19 was assessed using the Brief-Illness Perception Questionnaire (B-IPQ) (39), which evaluates cognitive, and emotional illness perception using a 10-point Likert scale with a total score ranging from 8 to 80. Observed scores were categorized into terciles (weak, moderate, strong). Item-level analysis was used to assess the perception of COVID-19 measured by the B-IPQ. The feeling of loneliness was assessed using the UCLA 3-item Loneliness Scale (3LS) (40) with a score range of 3–9. The presence of a feeling of loneliness was defined as a UCLA 3LS score ≥6. Resilience was assessed using the Connor-Davidson Resilience Scale (41) with a score range of 0–8. The presence of resilience was defined as low (score of 0–5), medium (score of 6–7), or high (score of 8). Resilient coping was assessed using the Brief Resilient Coping Scale (42) with a score range of 4–20. Resilient coping was defined as low (score of 4–13), medium (score of 14–16), or high (score of 17–20).

Compliance with COVID-19 lockdown measures was examined using a series of 4-point Likert scales: 1 (always), 2 (sometimes), 3 (seldom), and 4 (never). Spending quarantine alone or with others was measured using a multiple choice item that was transformed to a binary variable (alone/with others) (Supplementary Materials). Changes in nutrition, sleep length, and frequency of exercise were measured using self-reported ordinal items with levels “improved,” “without change” (referred to as “stable”), and “worsened.” The effect of COVID-19 lockdown measures on finances was examined using a 4-point ordinal item (with levels 1 “not at all,” 2 “just a little bit,” 3 “pretty much,” and 4 “extremely”) which were transformed into a 3-category variable with levels “none” (former level 1), “moderate” (former levels 2-3), and “extreme” (former level 4). Finally, the presence of selected diseases was measured using binary items.

### Data Analysis

Descriptive statistics were conducted for the socio-demographic variables and behavioral parameters. To test for age-related changes in mental health in response to the COVID-19 lockdown, participants were examined in three separate age groups, namely young (24–40 yr), middle-aged (41–55 yr), and older (56–68 yr) age groups. Age groups (based on age during the COVID-19 add-on study) were selected as a balance between an even distribution of respondents and adulthood developmental characteristics. Missing values were identified in baseline stress (*N* = 13) and depressive symptom (*N* = 19) data, representing 1.8 and 2.7% of the sample, respectively. No missing value imputation was performed, only cases with a complete pair of values were used in statistical analysis. The missing data were considered completely at random with no overlapping cases and no observable pattern in their distribution in relation to sex, age, or education. There were no significant differences in the mean scores of stress levels and the severity of depressive symptoms between participants with and without baseline missing values. A one sample chi-square test was used to assess the characteristics of the research sample. A Fisher's exact test was used to examine differences in compliance with COVID-19 lockdown measures. Normality of the data assessed using a Shapiro-Wilk test disclosed a violation of the normality rules. As a result, a McNemar's test was used to assess differences in prevalence of nominal stress levels and the presence of depressive symptoms. Changes of stress and depressive symptoms were calculated as a median of difference between repeated measures (during COVID-19 score minus prior to COVID-19 score). We used a non-parametric Wilcoxon signed-rank test for repeated measure differences between prior to COVID-19 and during COVID-19 lockdown levels of stress and depressive symptoms. Between-group differences (based on sex, age, etc.) in cross-sectional levels and longitudinal median differences of stress and depressive symptoms were examined using a Mann–Whitney *U*-test and a Kruskal–Wallis test with a Dunn-Bonferroni *post-hoc* test to correct for multiple comparisons. The respective effect size indicators were calculated and transformed to Pearson's r for a uniform evaluation of effect sizes. Significance was evaluated at an α = 0.05, all confidence intervals were set at the 95% level, and all testing was 2-sided. All observed values are presented as median and interquartile range [IQR] unless otherwise indicated. Data were analyzed using SPSS v.21 and the figures were generated in R v.3.6.3 (https://www.r-project.org/) with the ggplot2 (v.1.0.12) and pheatmap (v.2.3.3.0) packages.

### Ethical Consideration

The research protocol of the COVID-19 add-on study was approved by the Kardiovize study Internal Review Board as well as by the St. Anne's University Hospital ethics committee. Written informed consent was obtained from all participants of the COVID-19 add-on study.

## Results

### Demographics of the COVID-19 Population-Based Sample

The COVID-19 population-based sample consisted of 715 participants, among whom 379 (53%) were women and 336 (47%) were men, with a mean age of 46.12 (range, 24–68; SD, 10.94) (Table 2). The distribution of participants in the age groups was acceptably even. Participants were largely well-educated considering many of them completed university studies (347, 48.7%), followed by those with General Certificate of Secondary Education (GCSE) (274, 38.4%). Couples and small families represented approximately half of the population sample.

**Table 2:** Demographic characteristics of the COVID-19 general population-based sample.

	***N* (%)**	***P***
*N*	715	
Age (mean ±SD)	46.12 ± 10.94	
**Sex**		
Men	336 (47%)	0.11
Women	379 (53%)	
**Age groups**		
24–40 yrs	265 (37.1%)	<0.001
41–55 yrs	267 (37.3%)	
56–68 yrs	183 (25.6%)	
**Education**		
Without GCSE^a^	92 (12.9%)	<0.001
With GCSE^a^	274 (38.4%)	
University^b^	347 (48.7%)	
**Family members**		
1	100 (14.0%)	<0.001
2	256 (35.9%)	
3	145 (20.3%)	
4+	213 (29.8%)	

a *GCSE, General Certificate of Secondary Education*.

b *University education includes higher vocational school, bachelor, master, and doctoral degrees*.

### Compliance With COVID-19 Lockdown Measures

We first investigated how well the participants of the COVID-19 add-on study complied with the national lockdown measures imposed by the Czech government. To this end, we asked participants of the COVID-19 add-on study whether they always, sometimes, seldom, or never observed individual national lockdown measures. We found that 77.6, 75.7, and 51.6% of the participants “always” observed wearing a mask, increased hand hygiene, and respected the maximum of two people staying together in public places, respectively (Supplementary Table 1). Restriction of leaving home only when necessary (going to work, essential grocery, and medicine shopping), respecting 2 m social distancing, and reducing physical contact were “always” observed in 25.9, 33.6, and 30.8% and “sometimes” observed in 48, 57.5, and 55.9% of the participants, respectively. Women were statistically significantly more compliant in regard to all national lockdown measures compared to men. The older age group was statistically significantly more compliant in wearing a mask, in respecting 2 m social distancing, and in reducing physical contact compared with young and middle-aged adults. In regard to increased hand hygiene, respecting the maximum of two people staying together in public places, restricting leaving home only when necessary, and reducing physical contact, however, the older age group behaved similarly to the other age groups.

### Stress Levels During the COVID-19 Lockdown

**Figure 1:**
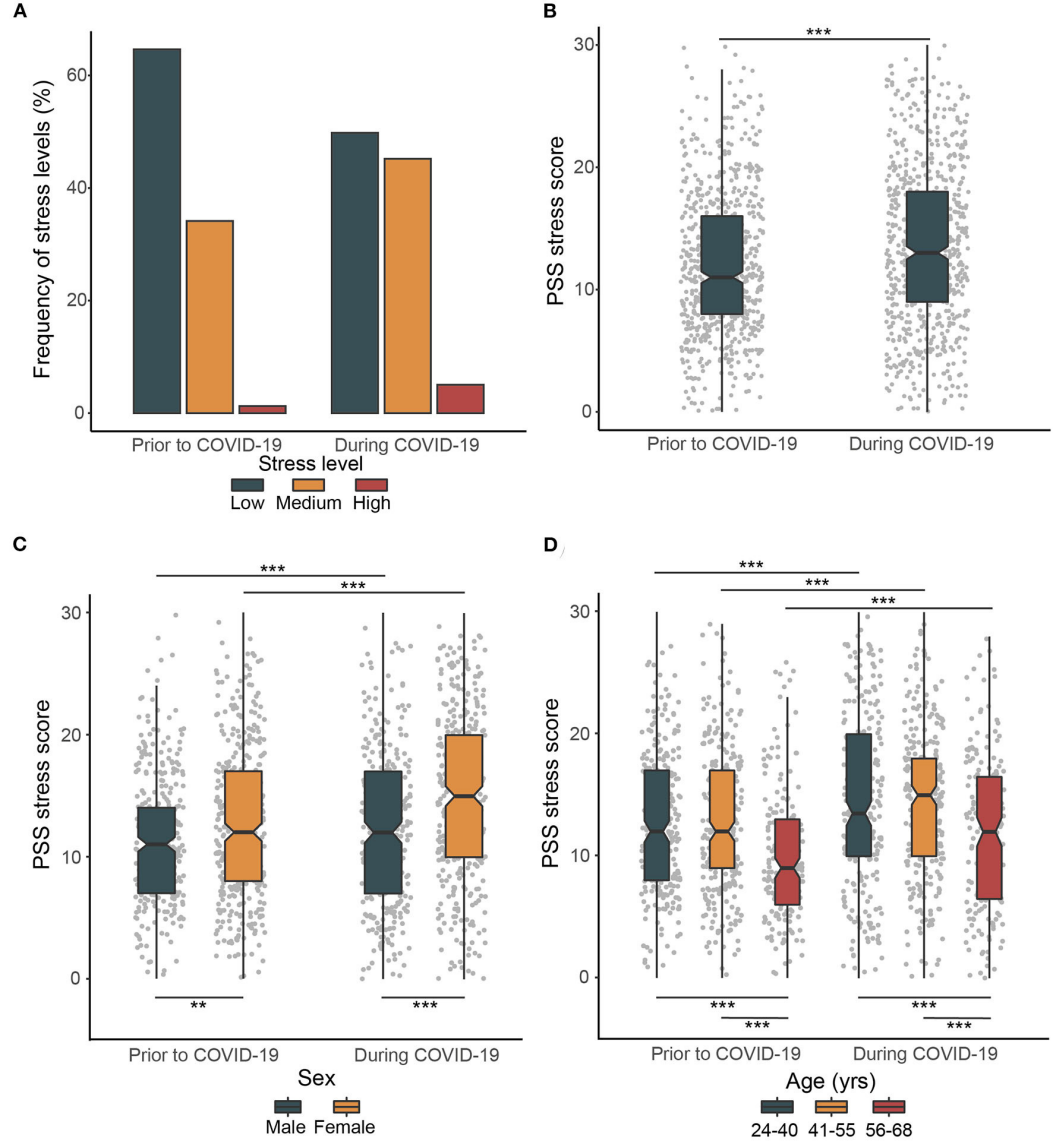
Prevalence of stress level prior to and during COVID-19 lockdown. **(A)** Frequency of stress levels prior to and during COVID-19 lockdown. **(B)** Changes in PSS stress score prior to and during COVID-19. **(C)** Sex differences in changes in stress levels prior to and during COVID-19. **(D)** Age group differences in changes in stress levels prior to and during COVID-19. The box plots with whiskers represent the median and the first and the third quartiles are extended by 1.5 times the interquartile range. Upper horizontal bars indicate significant differences in stress levels prior to and during COVID-19, lower horizontal bars indicate significant cross-sectional differences in stress levels between individual groups (**P < 0.01, ***P < 0.001).

We first measured stress that participants may have incurred during the COVID-19 lockdown. The prevalence of moderate to high stress amounted to 253 (35.4%, CI=32.5–39.7) and 359 participants (51.1%, CI=47.4–54.9) prior to and during the COVID-19 lockdown, respectively. The number of participants reporting moderate to high stress thus increased 1.4 times during the COVID-19 lockdown (*P* < 0.001) (Figure 1A). Accordingly, the PSS mean score also increased significantly in response to the COVID-19 lockdown (*P* < 0.001) (Figure 1B). This significant increase in stress during the COVID-19 lockdown was observed in both sexes (both *P* < 0.001), however, the observed surge in stress levels was significantly higher in women than in men (*P* = 0.01) (Figure 1C). Intriguingly, despite the fact that all age groups witnessed a significant and comparable increase in stress levels in response to the COVID-19 lockdown (*P* < 0.001), the older age group exhibited significantly lower stress levels prior to (*P* < 0.001) and during COVID-19 (*P* < 0.001) compared with the younger age groups (Figure 1D).

### Depressive Symptoms During COVID-19 Lockdown

**Figure 2:**
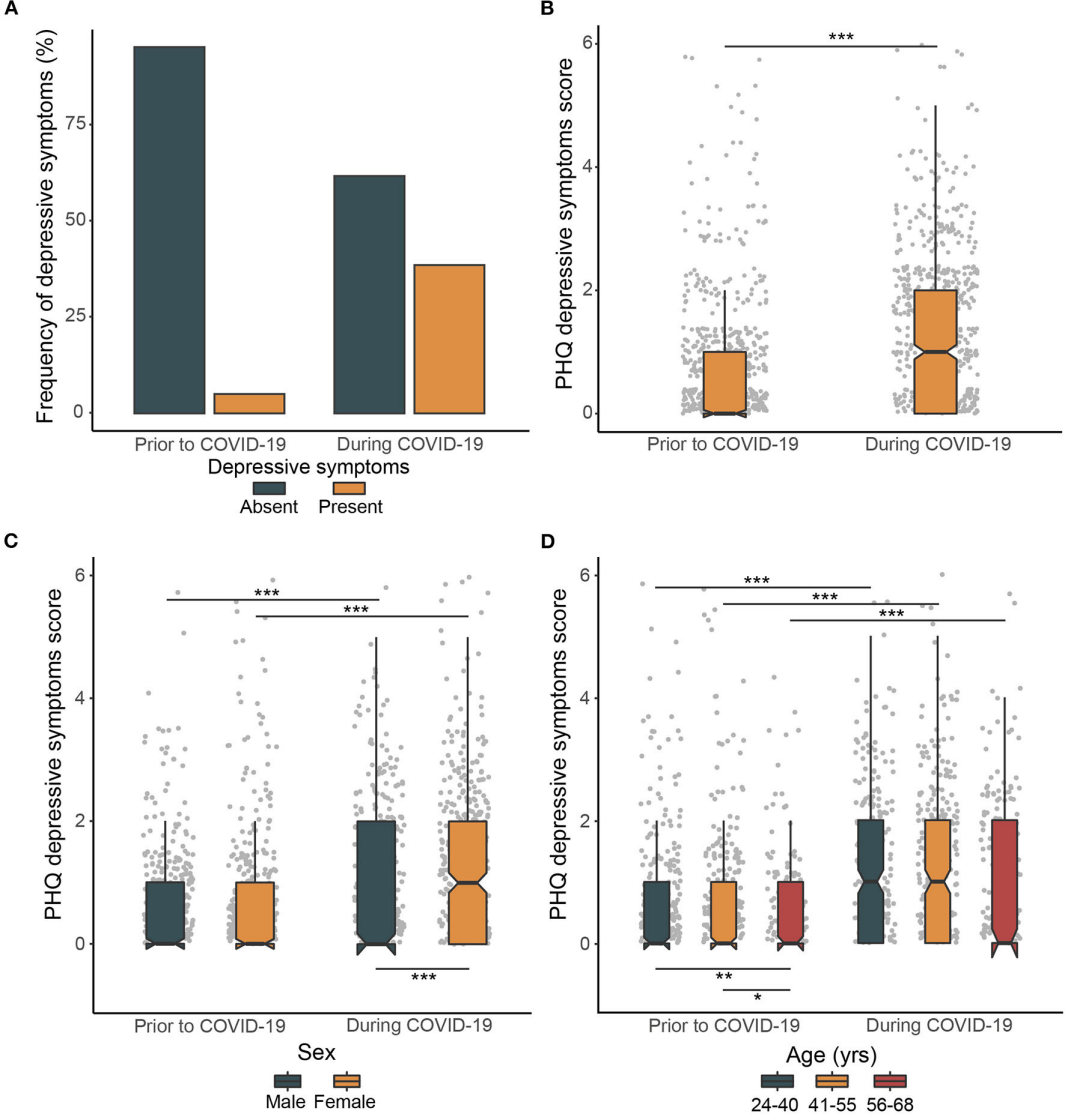
Prevalence of severity of depressive symptoms prior to and during COVID-19 lockdown. **(A)** Frequency of depressive symptoms prior to and during COVID-19 lockdown. **(B)** Changes in the PHQ depressive symptoms score prior to and during COVID-19. **(C)** Sex differences in changes in the severity of depressive symptoms prior to and during COVID-19. **(D)** Age group differences in changes in the severity of depressive symptoms prior to and during COVID-19. The box plots with whiskers represent the median and the first and the third quartiles are extended by 1.5 times the interquartile range. Upper horizontal bars indicate significant differences in severity of depressive symptoms prior to and during COVID-19, lower horizontal bars indicate significant cross-sectional differences in severity of depressive symptoms between individual groups (*P < 0.05, **P < 0.01, ***P < 0.001).

We next examined depressive symptoms prior to and during the COVID-19 lockdown. The prevalence of depressive symptoms amounted to 49 (7%, CI=5.3–9.2) and 269 cases (38.6%, CI=35.0–42.4) prior to and during the COVID-19 lockdown, respectively (Figure 2A). The number of participants reporting depressive symptoms thus increased 5.5 times during the COVID-19 lockdown compared with the pre-COVID-19 period (*P* < 0.001). Similarly, the severity of depressive symptoms also increased significantly during the COVID-19 lockdown (*P* < 0.001) (Figure 2B). This rise in depressive symptoms was present in both sexes (both *P* < 0.001), however, the observed increase in the severity of depressive symptoms was significantly higher in women than in men (*P* = 0.002) (Figure 2C). All age groups showed a significant and comparable increase in the severity of depressive symptoms in response to COVID-19 (all *P* < 0.001) with the older age group exhibiting a significantly lower severity of depressive symptoms prior to (*P* = 0.004), but not during the COVID-19 lockdown (*P* = 0.062) (Figure 2D).

### Risk Factors Associated With Increased Stress Levels and Depressive Symptoms

**Figure 3:**
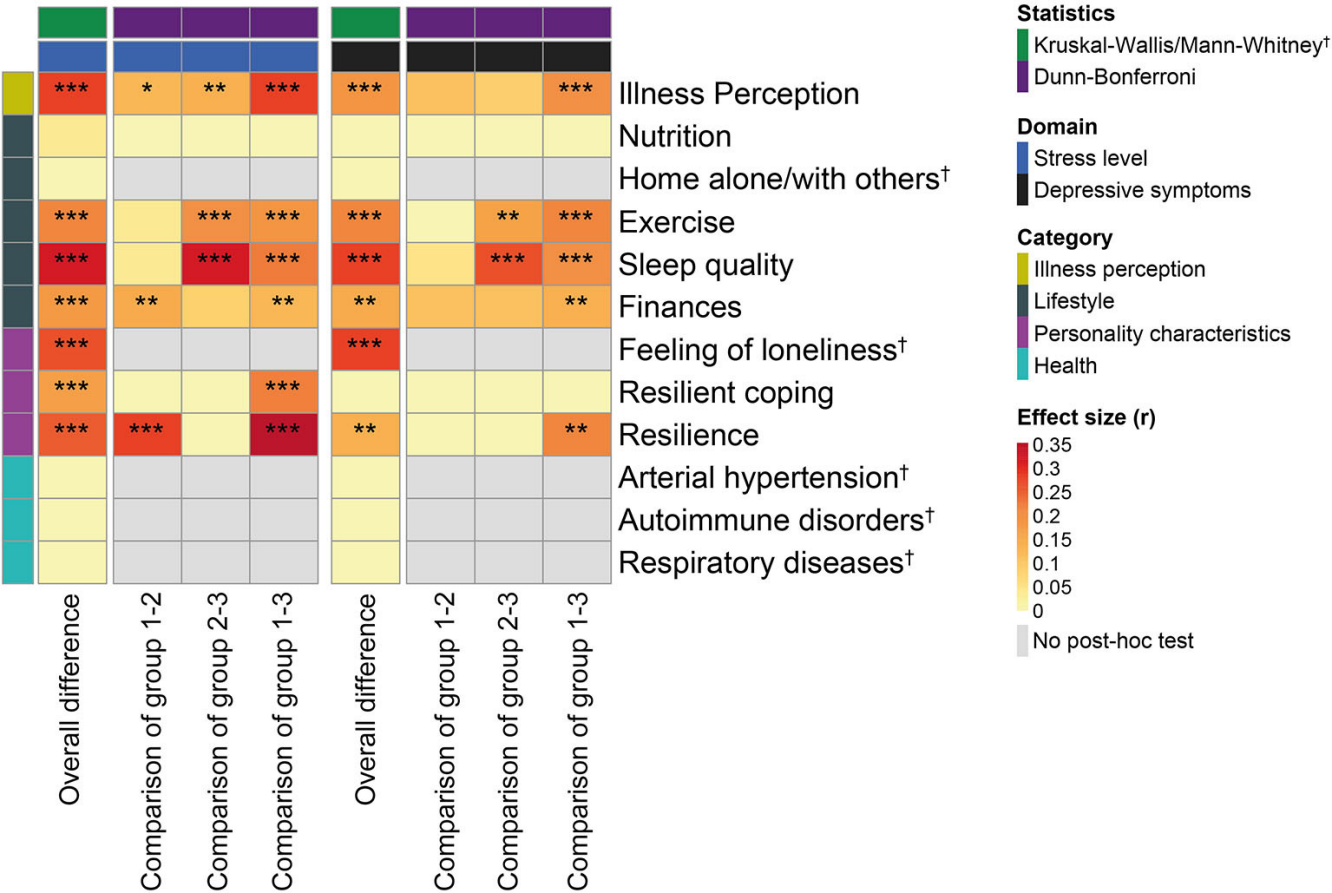
Association between stress levels, severity of depressive symptoms, and potential underlying risk factors during the COVID-19 lockdown. Heatmap showing Pearson's r effect sizes with levels of significance based on a Kruskal–Wallis/Dunn–Bonferroni test (with adjustment for multiple comparisons) and whenever appropriate a Mann–Whitney *U*-test (*P < 0.05, **P < 0.01, ***P < 0.001). Group comparisons refer to *post hoc* tests where for illness perception, finances, resilient coping and resilience groups 1, 2, and 3 correspond to minor, moderate, and significant response, respectively, while for nutrition, exercise, and sleep quality groups 1, 2, and 3 correspond to improved, unchanged, and worsened response, respectively.

In order to identify potential risk factors associated with the observed significant increase in stress levels and the severity of depressive symptoms in response to the COVID-19 lockdown, we investigated several aspects of illness perception, personality characteristics, lifestyle, and medical conditions. We first asked whether the perception of COVID-19 contributed to stress levels and depressive symptoms. The B-IPQ results showed that those who perceived COVID-19 as most threatening exhibited significantly higher stress levels and severity of depressive symptoms (*P* < 0.001) (Figure 3). Stress levels and severity of depressive symptoms were mostly affected by the general worry about COVID-19, the effect of COVID-19 on their emotional processing, the impact of COVID-19 on life, and the timeline of the COVID-19 risk (Supplementary Table 2). Moreover, the severity of depressive symptoms was to some degree also affected by the difficulties in understanding COVID-19 symptoms and mistrust in COVID-19 treatment options.

We next explored different personality characteristics in relation to changes in stress and depressive symptoms during the COVID-19 lockdown (Figure 3). The UCLA 3LS results revealed that a feeling of loneliness was associated with a significant increase in stress levels and severity of depressive symptoms in response to lockdown (*P* < 0.001). The lack of resilience measured using the Connor-Davidson Resilience scale also resulted in a significant increase in stress levels (*P* < 0.001) and severity of depressive symptoms (*P* = 0.007) during the COVID-19 lockdown. Non-adaptive coping strategies examined using the Brief Resilient Coping scale on the other hand, only produced a significant increase in stress levels (*P* = 0.001).

Social isolation and distancing represented a major change in lifestyle during the COVID-19 lockdown. As a result, we investigated the association between different lifestyle components and changes in stress and depressive symptoms during COVID-19 (Figure 3). We found that those who reported spending quarantine at home alone or with others both exhibited significantly increased stress levels and depressive symptoms (all *P* < 0.001). This indicated that social isolation did not play a role in changes to stress levels (*P* = 0.77) and depressive symptoms (*P* = 0.33) during the lockdown. Similarly, changes in nutrition during the COVID-19 lockdown were also not associated with increased stress and depressive symptoms (*P* = 0.25 and 0.37). In contrast, those who exercised less and reported poor sleep all demonstrated a significant increase in stress levels and depressive symptoms (all *P* < 0.001). Last, but not least, all those who reported that COVID-19 influenced their financial situation reported significantly increased stress levels and severity of depressive symptoms (all *P* < 0.001).

Considering the pathophysiology of COVID-19, which exploits ACE receptors to access respiratory cells and promotes a significant immune response, we last evaluated whether participants afflicted by arterial hypertension, respiratory diseases, or autoimmune disorders showed changes in stress levels and depressive symptoms in response to the COVID-19 lockdown (Figure 3). We found that none of the participants afflicted by arterial hypertension, respiratory diseases, or autoimmune disorders exhibited significant changes in stress levels (*P* = 0.26, 0.77, 0.87) and depressive symptoms (*P* = 0.87, 0.84, 0.18).

## Discussion

The main goal of this *ad hoc* study precipitated by the COVID-19 pandemic was to explore potential risk factors underlying mental distress in response to the COVID-19 lockdown. To this end, we first measured changes in stress levels and depressive symptoms longitudinally in a well-characterized population-based sample. We next searched for age-related changes and potential risk factors linked to the measured changes in stress levels and depressive symptoms during the COVID-19 lockdown.

Considering we planned to investigate changes in mental distress in response to the COVID-19 lockdown, we first asked whether participants of the COVID-19 add-on study complied with the government-imposed COVID-19 lockdown measures. We found that a large majority of the COVID-19 add-on study participants complied with the government-imposed lockdown measures comparable to COVID-19 lockdown compliance rates reported by others (43–45). However, we also learned that the COVID-19 add-on study participants demonstrated better compliance with some measures such as wearing a mask than with other measures such as the restriction of leaving home only when necessary. Our data also showed that women and the older age group demonstrated better compliance with government-imposed lockdown measures than men and younger adults.

Knowing that the COVID-19 add-on study participants complied satisfactorily with the government-imposed lockdown measures, we next measured the impact of COVID-19 lockdown on stress levels and the severity of depressive symptoms. Our measurements showed that COVID-19 lockdown resulted in a significant 1.4- and 5.5-fold increase in stress levels and depressive symptoms, respectively. The observed increase in stress levels and severity of depressive symptoms is consistent with reported cross-sectional (26, 46, 47) and longitudinal (15, 17, 19) general population studies. In agreement with previous reports (48, 49), we found a more pronounced impact of the COVID-19 lockdown on the mental health of women.

All age groups exhibited a significant and comparable increase in mental distress in response to the COVID-19 lockdown, with the older age group showing generally lower levels of mental distress. These longitudinal findings extend our current understanding of the interaction between age and COVID-19 (50–52) by showing that all age groups exhibited the same susceptibility to COVID-19-induced mental distress. Cross-sectional analysis of these findings indicated a benefit of the generally lower mental distress in the older group despite the same susceptibility to COVID-19-induced mental distress compared to other age groups. This may be due to the association of greater worries about studies, job security, and financial stability with the younger age group, and the richer life experiences and reduced life expectations in the older group (53–55).

Longitudinal measurement of the increase in stress and depressive symptoms in response to the COVID-19 lockdown enabled us to search for potential risk factors linked to the observed changes in mental health. We identified illness perception, several personality characteristics, and lifestyle components, but not pre-existing medical conditions, as potential risk factors. In brief, those who perceived COVID-19 as emotionally threatening exhibited the highest significant increase in stress levels and severity of depressive symptoms. Similarly, a feeling of loneliness was identified as the most significant risk factor translating into a major surge in stress levels and severity of depressive symptoms in response to the COVID-19 lockdown. This finding is in agreement with recent reports (21, 56) and further corroborates the intimate link between the feeling of loneliness and mental distress (57–60). On a different note, we also identified the positive effect of resilience and resilient coping on COVID-19 lockdown-induced mental distress (32, 61). This finding in particular, may be well-suited for interventions designed to decrease and better control mental distress in response to public health emergencies. In contrast to other changes in lifestyle, such as exercising and sleep, our study found no association between mental distress and spending the government-imposed quarantine alone or with others. This is inconsistent with recent cross-sectional studies (62–64), but could also be the consequence of suboptimal compliance with observing the restriction of leaving home only when necessary. In summary, although further research is needed to demonstrate causality, we here identified several potential risk factors associated directly with the surge in mental distress in response to the COVID-19 lockdown.

There are several major strengths of our study. First, we measured stress and depression longitudinally in a well-characterized population sample, which contrasts with convenience or probabilistic sampling using national surveys. Second, we thoroughly investigated the role of age in the observed surge in mental distress in response to the COVID-19 lockdown. And third, we critically investigated potential risk factors based on the longitudinally measured increase in stress levels and severity of depressive symptoms in response to the COVID-19 lockdown using an extensive battery of measurement instruments.

Our study has also its limitations. First, the population sample is rather small compared to some recently reported studies (17, 19, 65). Second, only 40% of the participants of the Kardiovize study accepted the electronic invitation to participate in the COVID-19 add-on study. Although one may envision many reasons for the observed low enrolment rate, it could well be that those exhibiting the highest mental distress in response to COVID-19 were actually those who most commonly declined participation in the COVID-19 add-on study. In this case, our measurements are an underestimation of the actual impact of COVID-19 on mental health. And third, the Kardiovize and COVID-19 add-on study participants in general mostly represented the urban population with a higher education compared with the rural population.

In conclusion, this study provides repeated measure-based evidence of an increase in stress levels and the severity of depressive symptoms in a sample of the general population during the COVID-19 lockdown. Importantly, older participants showed the same degree of susceptibility to the COVID-19-induced mental distress as the younger group, but benefited from generally lower mental distress. Finally, our study identified illness perception, a feeling of loneliness, resilience and resilient coping, and several lifestyle changes as potential risk factors underlying the observed surge in mental distress in response to the COVID-19 lockdown. Observed mental distress and many of the identified risk factors can be prevented, diagnosed, and treated, although such interventions need to be tailored to the public health emergency setting. More intense and better organized approaches to mental distress and the underlying risk factors in the general population need to be integrated into global public health policies to protect mental health during future pandemics.

## Data Availability Statement

The raw data supporting the conclusions of this article will be made available by the authors, without undue reservation.

## Ethics Statement

The studies involving human participants were reviewed and approved by St. Anne's University Hospital ethics committee and Kardiovize study Internal Review Board. The patients/participants provided their written informed consent to participate in this study.

## Author Contributions

GS and JN had full access to all the data in the study, take responsibility for the integrity of the data, the accuracy of the data analysis, and wrote the draft of the manuscript. GS conceived the idea of this study. JN made the statistical analysis. All authors contributed to the critical revision of the manuscript for important intellectual content.

## Conflict of Interest

The authors declare that the research was conducted in the absence of any commercial or financial relationships that could be construed as a potential conflict of interest.
