# Supplementary material for: Risk Factors Underlying COVID-19 Lockdown-Induced Mental Distress
Source: Front Psychiatry. 2020 Dec 21;11:603014. doi: 10.3389/fpsyt.2020.603014 (PMC7793642; doi:10.3389/fpsyt.2020.603014)
Supplement: Supplementary file 1 [file Table_1.DOCX]

Supplementary Material

# Supplementary Figures and Tables


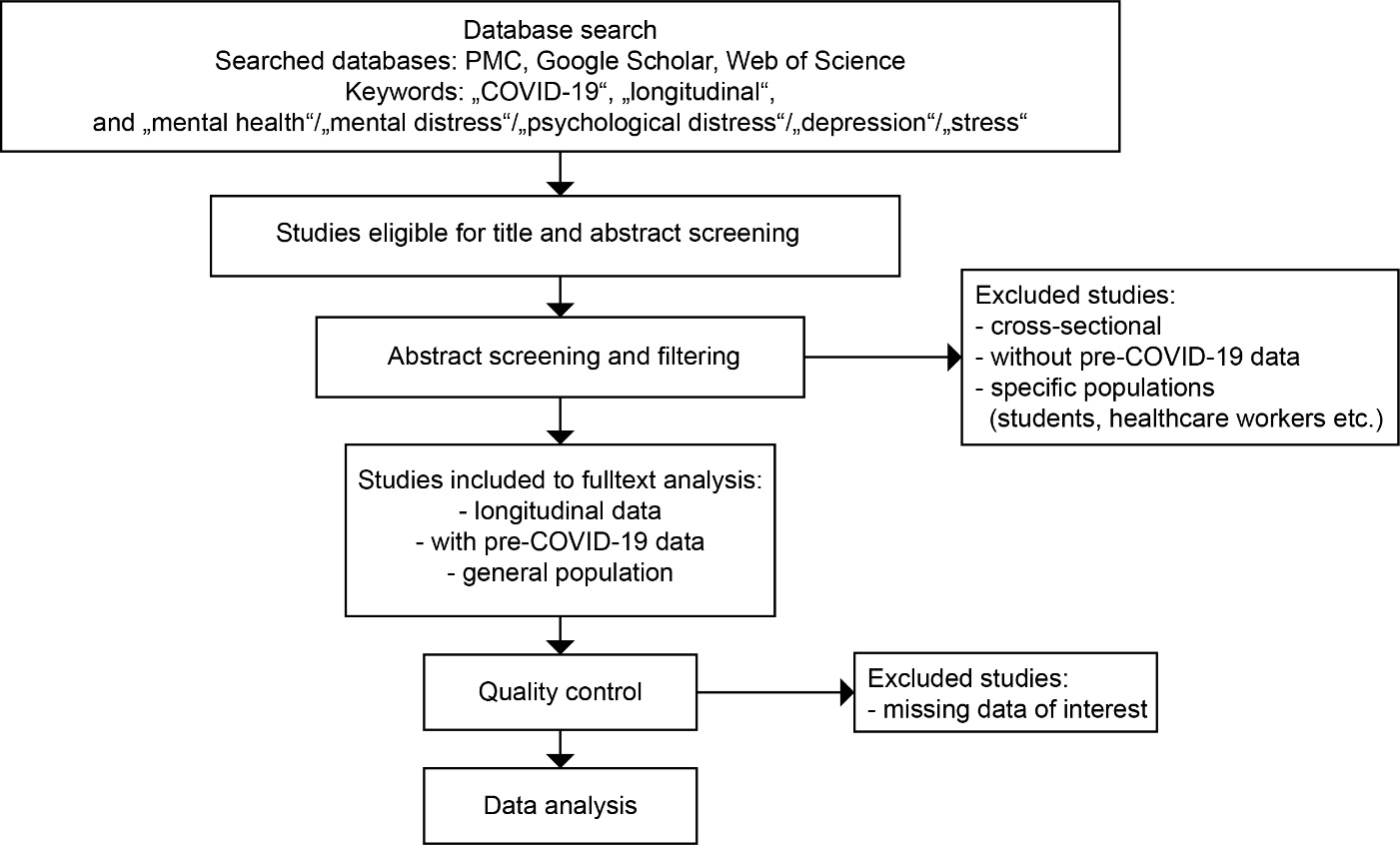


**Supplementary Figure 1.** Flow-chart depicting the selection process of the rapid review of current longitudinal studies of the impact of COVID-19 on mental health.


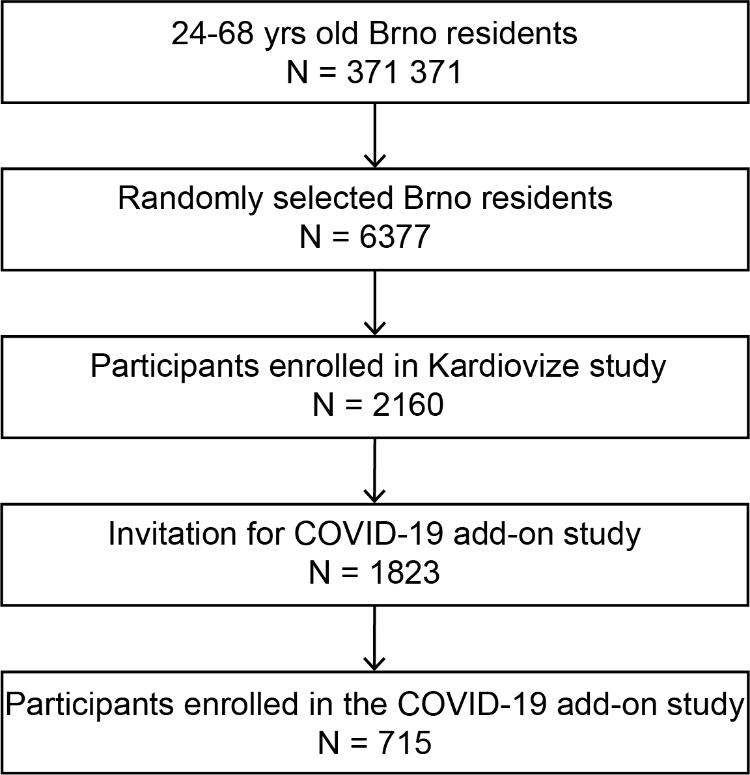


**Supplementary Figure 2.** Flow-chart depicting selection process of the participants included in the COVID-19 add-on study.

**Supplementary Table 1**. Compliance with the Czech Republic COVID-19 lockdown measures^a^

|  | **Always** | **Sometimes** | **Seldom** | **Never** | **P^b^** |
| --- | --- | --- | --- | --- | --- |
| **Wearing a mask** |  |  |  |  |  |
| Males | 238 (70.8%) | 89 (26.5%) | 8 (2.4%) | 1 (0.3%) |  |
| Females | 317 (83.6%) | 56 (14.8%) | 6 (1.6%) | 0 (0%) | <0.001 |
| 24-40 yrs | 192 (72.5%) | 65 (24.5%) | 7 (2.6%) | 1 (0.4%) |  |
| 41-55 yrs | 201 (75.3%) | 61 (22.8%) | 5 (1.9%) | 0 (0%) |  |
| 56-68 yrs | 162 (88.5%) | 19 (10.4%) | 2 (1.1%) | 0 (0%) | 0.001 |
| *Total* | *555 (77.6%)* | *145 (20.3%)* | *14 (2%)* | *1 (0.1%)* |  |
| **Increased hand hygiene** |  |  |  |  |  |
| Males | 224 (66.7%) | 95 (28.3%) | 16 (4.8%) | 1 (0.3%) |  |
| Females | 317 (83.6%) | 49 (12.9%) | 10 (2.6%) | 3 (0.8%) | <0.001 |
| 24-40 yrs | 191 (72.1%) | 58 (21.9%) | 15 (5.7%) | 1 (0.4%) |  |
| 41-55 yrs | 203 (76%) | 55 (20.6%) | 8 (3%) | 1 (0.4%) |  |
| 56-68 yrs | 147 (80.3%) | 31 (16.9%) | 3 (1.6%) | 2 (1.1%) | 0.16 |
| *Total* | *541 (75.7%)* | *144 (20.1%)* | *26 (3.6%)* | *4 (0.6%)* |  |
| **Restriction of leaving home** |  |  |  |  |  |
| Males | 78 (23.2%) | 147 (43.8%) | 82 (24.4%) | 29 (8.6%) |  |
| Females | 107 (28.2%) | 196 (51.7%) | 56 (14.8%) | 20 (5.3%) | 0.001 |
| 24-40 yrs | 64 (24.2%) | 130 (49.1%) | 55 (20.8%) | 16 (6%) |  |
| 41-55 yrs | 72 (27%) | 125 (46.8%) | 52 (19.5%) | 18 (6.7%) |  |
| 56-68 yrs | 49 (26.8%) | 88 (48.1%) | 31 (16.9%) | 15 (8.2%) | 0.90 |
| *Total* | *185 (25.9%)* | *343 (48%)* | *138 (19.3%)* | *49 (6.9%)* |  |
| **2m social distancing** |  |  |  |  |  |
| Males | 87 (25.9%) | 213 (63.4%) | 35 (10.4%) | 1 (0.3%) |  |
| Females | 153 (40.4%) | 198 (52.2%) | 28 (7.4%) | 0 (0%) | <0.001 |
| 24-40 yrs | 74 (27.9%) | 157 (59.2%) | 34 (12.8%) | 0 (0%) |  |
| 41-55 yrs | 85 (31.8%) | 159 (59.6%) | 22 (8.2%) | 1 (0.4%) |  |
| 56-68 yrs | 81 (44.3%) | 95 (51.9%) | 7 (3.8%) | 0 (0%) | <0.001 |
| *Total* | *240 (33.6%)* | *411 (57.5%)* | *63 (8.8%)* | *1 (0.1%)* |  |
| **Reducing physical contact** |  |  |  |  |  |
| Males | 87 (25.9%) | 191 (56.8%) | 51 (15.2%) | 7 (2.1%) |  |
| Females | 133 (35.1%) | 209 (55.1%) | 31 (8.2%) | 6 (1.6%) | 0.005 |
| 24-40 yrs | 73 (27.5%) | 149 (56.2%) | 39 (14.7%) | 4 (1.5%) |  |
| 41-55 yrs | 69 (25.8%) | 161 (60.3%) | 31 (11.6%) | 6 (2.2%) |  |
| 56-68 yrs | 78 (42.6%) | 90 (49.2%) | 12 (6.6%) | 3 (1.6%) | 0.002 |
| *Total* | *220 (30.8%)* | *400 (55.9%)* | *82 (11.5%)* | *13 (1.8%)* |  |
| **Max 2 people together in public** |  |  |  |  |  |
| Males | 148 (44%) | 127 (37.8%) | 44 (13.1%) | 17 (5.1%) |  |
| Females | 221 (58.3%) | 124 (32.7%) | 31 (8.2%) | 3 (0.8%) | <0.001 |
| 24-40 yrs | 129 (48.7%) | 102 (38.5%) | 26 (9.8%) | 8 (3%) |  |
| 41-55 yrs | 130 (48.7%) | 99 (37.1%) | 28 (10.5%) | 10 (3.7%) |  |
| 56-68 yrs | 110 (60.1%) | 50 (27.3%) | 21 (11.5%) | 2 (1.1%) | 0.085 |
| *Total* | *369 (51.6%)* | *251 (35.1%)* | *75 (10.5%)* | *20 (2.8%)* |  |

^a^Data are presented as number of participants (%).

^b^Fisher’s exact test of differences in compliance between sex and age groups.

**Supplementary Table 2**. Association between individual items of the Brief-Illness Perception Questionnaire and changes in stress level and severity of depressive symptoms^a^ during the COVID-19 lockdown

|  | **Median difference of PHQ and PSS scores**  **during COVID–19^b^** | | | **Kruskal-Wallis test** | **Pairwise comparisons p-values^c^** | | |
| --- | --- | --- | --- | --- | --- | --- | --- |
| **B-IPQ levels** | **Weak (W)** | **Moderate (M)** | **Strong (S)** | **P** | **W-M** | **M-S** | **W-S** |
| Stress levels (N=702)^d^ |  |  |  |  |  |  |  |
| Impact on life^f^ | 1 [-3–5] | 1 [-3–6] | 4 [-1–9] | <0.001 | 0.54 | 0.001 | <0.001 |
| Timeline of illness risk^f^ | 0.5 [-5–5] | 2 [-2–6] | 3 [-2–7] | 0.001 | 0.009 | 0.39 | 0.001 |
| Control over getting ill^f^ | 1.5 [-2.5–7] | 2 [-2–7] | 1 [-3–6] | 0.23 |  |  |  |
| Trust in treatment^f^ | 2.5 [-1.5–8] | 2 [-2–7] | 1 [-3–6] | 0.14 |  |  |  |
| Symptoms perception^f^ | 1 [-5–5] | 2 [-2–7] | 2 [-2–7] | 0.10 |  |  |  |
| Worries about Covid-19^f^ | 0 [-4–5] | 2 [-1–7] | 4 [0–9] | <0.001 | 0.001 | 0.009 | <0.001 |
| Understanding Covid-19^f^ | 2 [-3–8] | 2 [-2–7] | 1 [-4–7] | 0.35 |  |  |  |
| Effect on emotions^f^ | 0 [-4–4] | 3 [-1–7] | 7 [2–11] | <0.001 | <0.001 | <0.001 | <0.001 |
| Severity of depressive symptoms (N=696)^§e^ |  |  |  |  |  |  |  |
| Impact on life^f^ | 0 [0–1] | 0 [0–1] | 1 [0–2] | 0.001 | 0.14 | 0.13 | <0.001 |
| Timeline of illness risk^f^ | 0 [0–1] | 0 [0–1] | 0 [0–2] | 0.04 | 0.18 | 1.000 | 0.03 |
| Control over getting ill^f^ | 0 [0–1] | 0 [0–1] | 0 [0–1] | 0.59 |  |  |  |
| Trust in treatment^f^ | 0 [0–1] | 0 [0–2] | 0 [0–1] | 0.04 | 0.21 | 0.76 | 0.03 |
| Symptoms perception^f^ | 0 [0–1] | 0 [0–2] | 0 [0–1] | 0.04 | 0.23 | 1.000 | 0.04 |
| Worries about Covid-19^f^ | 0 [0–1] | 0 [0–2] | 1 [0–2] | 0.06 |  |  |  |
| Understanding Covid-19^f^ | 0 [0–1] | 0 [0–1] | 0 [0–1] | 0.73 |  |  |  |
| Effect on emotions^f^ | 0 [0–1] | 0.5 [0–2] | 1 [0–3] | <0.001 | <0.001 | 0.003 | <0.001 |

^a^PHQ = Patient Health Questionnaire (depressive symptoms); PSS = Perceived Stress Scale.

^b^Values presented as median [IQR]

^c^P-values of Dunn-Bonferroni test adjusted for multiple comparison (for significant Kruskal-Wallis tests).

# Kardiovize COVID-19 add-on study e-questionnaire

1. What is your current weight?
2. How many cigarettes do you smoke per day? If you are a non-smoker, please, fill 0.
3. What is your current family situation?
   1. Living in a relationship with children
   2. Living in a relationship without children
   3. Monoparental household (children living with one parent)
   4. Living alone
   5. Other (please, specify)
4. How many children do you have?
   1. None
   2. One child
   3. Two children
   4. Three or more children
5. Who are you spending your time with during the quarantine? (multiple choice)
   1. No one
   2. With my partner or spouse
   3. With my children
   4. With other family members
   5. With someone outside my own family
6. During the last 14 days, how often have you been actively and specifically seeking information about the current situation regarding the COVID-19 pandemic and related measures?
   1. Never
   2. Less than once per week
   3. 1-2 times per week
   4. 2-3 times per week
   5. Approximately once per day
   6. Many times per day
7. Does the COVID-19 state of emergency affect your financial situation?
   1. Not at all
   2. Just a little
   3. Pretty much
   4. Extremely
8. How much does the current COVID-19 pandemic affect your work life? (multiple choice)
   1. The pandemic does not affect my work life/I am currently not working
   2. I have more work than usual
   3. I have less work than usual
   4. I work from home
   5. I changed my job/my job duties or position changed
   6. I stayed home because of kids or family member
   7. I lost my job
9. How many individual private (not work-related) social contacts (phone, SMS, Skype, WhatsApp, email, ...) have you had in the past 7 days?
   1. None, I am without social contacts
   2. 1 to 3 contacts
   3. 4 -7 contacts
   4. 8 -14 contacts
   5. 15 and more contacts
10. How many individual work-related social contacts (phone, SMS, Skype, WhatsApp, email, ...) have you had in the past 7 days?
    1. None
    2. 1 to 3 contacts
    3. 4 -7 contacts
    4. 8 -14 contacts
    5. 15 and more contacts
11. Has your sleep quality changed in the past 14 days?
    1. it got better
    2. it did not change
    3. it got worse
12. Has the length of your sleep changed (average per day)?
    1. sleep time has increased
    2. sleep time did not change
    3. sleep time has decreased

How often have you exercised in the past 14 days? Write down how many hours per week have you spent performing specific exercises, if zero time, fill in 0.

1. Low intensity exercise (e.g. walking):
2. High intensity exercise (e.g. running):
3. Body building:
4. Stretching:
5. Has the frequency of how often you exercise changed over the past 14 days?
   1. the frequency has increased
   2. the frequency has not changed
   3. the frequency has decreased
6. How many times per week did you go out from your home (work, shop, nature, etc.) in the past 14 days?
   1. Never
   2. 1-2 times per week
   3. 3-5 times per week
   4. Almost every day

**How do you comply with the government-imposed COVID-19 lockdown measures?**

1. Are you wearing a mask?
   1. Always
   2. Sometimes
   3. Seldom
   4. Never
2. How often are you washing or disinfecting your hands?
   1. Always
   2. Sometimes
   3. Seldom
   4. Never
3. How often have you respected restriction of leaving home only when absolutely necessary?
   1. Always
   2. Sometimes
   3. Seldom
   4. Never
4. How often have you respected the 2-meter social distancing?
   1. Always
   2. Sometimes
   3. Seldom
   4. Never
5. How often have you respected the ban of physical contact with other people?
   1. Always
   2. Sometimes
   3. Seldom
   4. Never
6. How often have you respected that only two people can be in closer contact in public places?
   1. Always
   2. Sometimes
   3. Seldom
   4. Never
7. When do you think the life will get back to normal in the Czech Republic*? Please indicate the number of months.*
8. How many days did you spend in quarantine?
   *If you have not been in quarantine, please fill in 0.*
   1. because of contact with a person with confirmed COVID-19 infection:
   2. because returning from a COVID-19 high risk country:
   3. because being tested positive for COVID-19:
9. Have you fell ill with COVID-19?
   1. Yes
   2. No
10. What symptoms or signs of COVID-19 have you manifested? (multiple choice)
    1. Fever
    2. Running nose and cough
    3. Emphysema
    4. Pneumonia
    5. Loss of taste and smell
    6. Headache and dizziness
    7. Nausea, vomiting, diarrhea
    8. Weakness, joints and muscle pain
    9. No symptoms or signs

**In the following section we will ask you about your health.**

1. Have you been treated for arterial hypertension?
   1. Yes
   2. No
2. If yes, please provide the names of the medications and the dosage to treat arterial hypertension:
3. Have you been diagnosed with diabetes mellitus type I?
   1. Yes
   2. No
4. If yes, please provide the names of the medications and the dosage to treat diabetes mellitus type I:
5. Have you been diagnosed for diabetes mellitus type II?
   1. Yes
   2. No
6. If yes, please provide the names of the medications and the dosage to treat diabetes mellitus type II:
7. Have you been diagnosed with a disease of the respiratory tract (asthma, etc.)?
   1. Yes
   2. No
8. If yes, please provide the names of the medications and the dosage to treat the respiratory track disease:
9. Have you been diagnosed with any of the following immune disorders?
   1. Inflammatory bowel disease (e.g. ulcerative colitis or Crohn's disease)
   2. Rheumatoid arthritis
   3. Multiple sclerosis
   4. Bone marrow transplant
   5. Organ transplant and immunosuppressive therapy
   6. Cancer treated with chemotherapy or radiotherapy
   7. None
10. If yes, please provide the names of the medications and the dosage to treat these disorders:
11. Have you been diagnosed with an allergy or atopic eczema?
    1. Yes
    2. No
12. Are you currently taking medications containing corticosteroid (e.g. Decamed, Medrol, Depo-Medrol, Dexamethasone, Hydrocortisone, Fortecortin, Methycetone, Fludrocortisone)?
    1. Yes
    2. No
13. Are you currently taking medications containing Hydrochloroquine (e.g. Plaquenil)?
    1. Yes
    2. No
